# Tips and tricks to achieve osteotomy healing and prevent refracture after ulnar shortening osteotomy

**DOI:** 10.1186/s13018-021-02266-z

**Published:** 2021-02-04

**Authors:** Jong Woo Kang, Soo Min Cha, Sang-gyun Kim, In Cheul Choi, Dong Hun Suh, Jong Woong Park

**Affiliations:** 1 Department of Orthopedic Surgery, Korea University Anam Hospital, Korea University College of Medicine, 73, Goryeodae-ro, Seongbuk-gu, Seoul, 02841 South Korea; 2 Department of Orthopedic Surgery, Regional Rheumatoid and Degenerative Arthritis Center, Chungnam National University Hospital, Chungnam National University School of Medicine, Daejeon, Korea; 3 Department of Orthopedic Surgery, Anam Hospital, College of Medicine, Korea University, Seoul, Korea

**Keywords:** Osteotomy, Union, Consolidation, Healing, Ulnar impaction syndrome

## Abstract

**Background:**

Parallel osteotomy is essential for favorable osteotomy reduction and healing and technically challenging during diaphyseal ulnar shortening osteotomy (USO). This study aimed to evaluate the advantages of guided osteotomy for parallel osteotomy and reduction osteotomies, healing over freehand osteotomy. It also aimed to identify surgical factors affecting healing after diaphyseal USO.

**Methods:**

Between June 2005 and March 2016, 136 wrists that had undergone diaphyseal USO for ulnar impaction syndrome (UIS) were evaluated. The wrists were divided into two groups according to the osteotomy technique (group 1: freehand osteotomy, 74 wrists; group 2: guided osteotomy, 62 wrists). The osteotomy reduction gap and time to osteotomy healing (union and consolidation) were compared between the groups. A multiple regression test was performed to identify the surgical factors affecting healing. The cut-off length of the reduction gap to achieve osteotomy union on time and the cut-off period to decide the failure of complete consolidation were statistically calculated.

**Results:**

The baseline characteristics did not differ between the two groups. The osteotomy reduction gap and time to osteotomy union, and complete consolidation were shorter in group 2 than in group 1 (*p* = 0.002, < 0.001, 0.002). The osteotomy reduction gap was a critical surgical factor affecting both time to osteotomy union and complete consolidation (*p* < 0.001, < 0.001). The use of a dynamic compression plate affected only the time to complete consolidation (*p* < 0.001). The cut-off length of the osteotomy reduction gap to achieve osteotomy union on time was 0.85 mm. The cut-off period to decide the failure of complete consolidation was 23.5 months after osteotomy.

**Conclusions:**

The minimal osteotomy reduction gap was the most important for timely osteotomy healing in the healthy ulna, and guided osteotomy was beneficial for reducing the osteotomy reduction gap.

## Introduction

Since Milch [1] first described distal diaphyseal ulnar shortening osteotomy (USO) in 1941, it has been the most popular surgical procedure for ulnar impaction syndrome (UIS) [2–7]. The clinical outcomes of USO are also satisfactory [2, 4, 6, 7]. Despite its good clinical outcomes, its critical shortcomings, including nonunion or delayed union following the procedure, have variable incidence (0–12.7%) and remain unresolved [2, 4, 8].

Healing after USO is affected by multiple factors that are both patients- and surgery-related [9]. The representative and well-known patient-related risk factors for nonunion or delayed union include age, low bone mineral density, obesity, diabetes, thyroid disease, smoking, and alcohol consumption [9–14]. Studies on surgical risk factors are limited, and the factors have not been identified [9]. Since surgical risk factors can be controlled by the surgeon while patient-related factors cannot be resolved entirely, identifying and avoiding surgical risk factors are critical in preventing or overcoming nonunion or delayed union.

This study aimed to evaluate whether guided osteotomy using a dedicated USO system is beneficial for parallel and reduction osteotomies and healing after USO (union and complete consolidation [disappearance of any trace of osteotomy]) compared to conventional freehand osteotomy and to identify the surgical factors affecting healing after USO.

## Methods

### Patients

This study was approved by the local institutional review board, and informed consent was obtained from all enrolled patients (2018AS0050). The study protocol conformed to the ethical guidelines of the 1975 Declaration of Helsinki. Of the 212 wrists that had undergone USO for idiopathic UIS between June 2005 and March 2016, we selected 136 wrists (124 patients, right wrist: 68, left wrist: 68, both wrists: 12) that met our inclusion and exclusion criteria and reviewed them retrospectively. The inclusion criteria included patients with idiopathic UIS who underwent USO and postoperative follow-up for a minimum of 2 years. Patients with a trauma history and/or comorbidities, such as diabetes, thyroid or parathyroid disease, renal disease, congenital metabolic bone disease, rheumatoid arthritis, severe obesity, low bone density, and patients on glucocorticoid therapy, or with a smoking or alcohol consumption history less than 1 month before USO were excluded avoiding false conclusions on the osteotomy healing.

**Fig. 1:**
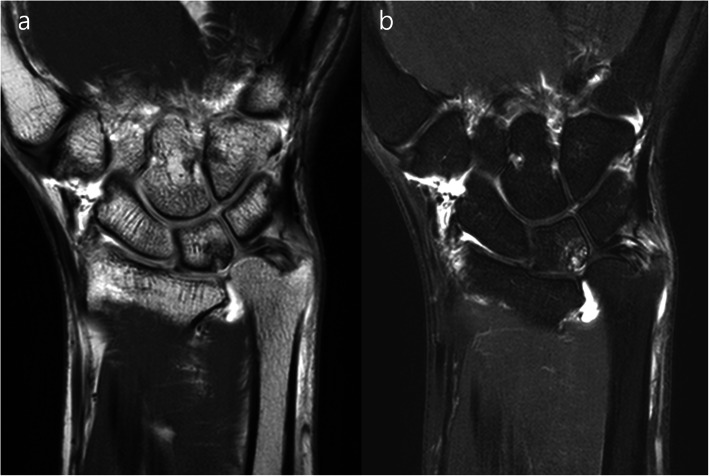
The magnetic resonance images of ulnar impaction syndrome (**a** T1-weighted image, **b** T2-weighted image on the coronal). They typically contain degenerations in an ulnocarpal joint (TFCC wear or perforation, perforation of a lunotriquetral ligament, and chondromalacia or cartilage denudation or bony edema or erosion of lunate or triquetrum or ulnar head)

The diagnostic criteria for UIS included symptoms of ulna-sided wrist pain that aggravated with pronation and ulnar deviation of the wrist, pain during the ulnar stress test, tenderness in the ulnocarpal joint, positive ulnar variance in pronated-grip radiographs during either neutral rotation or cystic changes in the lunate or triquetrum or ulnar head, and degenerations of ulnocarpal joint in magnetic resonance imaging (Fig. 1) [15].

### Study design

**Fig. 2:**
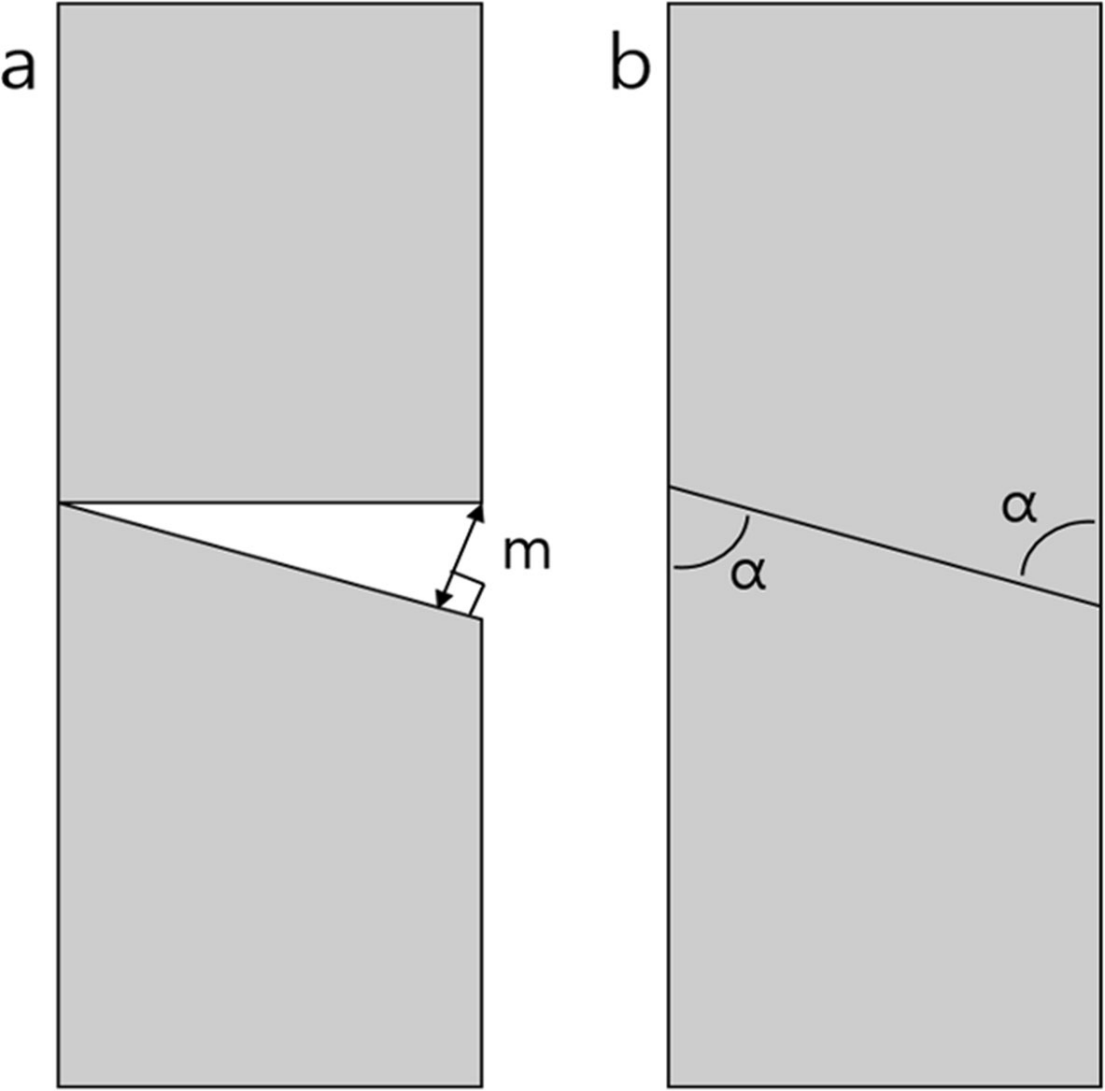
**a** The osteotomy reduction gap (m) is measured along with the longest distance between two surfaces of the osteotomy. **b** The osteotomy direction was classified as transverse or oblique osteotomy according to the acute angle (α) between the longitudinal axis and the osteotomy surface

For comparing the effect of the osteotomy technique on parallel osteotomy and osteotomy healing, the wrists were divided into two groups according to the osteotomy technique (group 1: freehand osteotomy, 74 wrists; group 2: guided osteotomy, 62 wrists). The osteotomy reduction gap (the longest distance between two osteotomized surfaces after osteotomy reduction, (Fig. 2a), time to osteotomy union, and time to complete osteotomy consolidation were compared between the groups.

For identifying the surgical factors affecting the time to osteotomy union and complete consolidation, we performed multiple regression tests for the direction of osteotomy, the gap after reduction osteotomy, length of ulnar shortening, usage of a lag screw, and plate type used for osteotomy fixation. Additionally, the cut-off length of the osteotomy reduction gap to obtain union within 6 months and the maximum period to achieve complete consolidation were statistically calculated.

### Surgery and postoperative rehabilitation

Distal diaphyseal USO was performed by two experienced surgeons under general anesthesia or brachial plexus block. Both surgeons used the same criteria to diagnose idiopathic UIS and the same surgical techniques. The periosteum of the ulna was not stripped during surgery to preserve the local blood supply around the osteotomy site. In group 1, freehand osteotomy was performed according to the previously described technique, and the osteotomy was fixed with a 3.5-mm limited contact-dynamic (LC-DCP; Dupuy-Synthes, Paoli, PA, USA) or locking (LCP; Dupuy-Synthes, Paoli, PA, USA) compression plate, after manual reduction [6]. In group 2, guided osteotomy was performed with a 2.7-mm (Dupuy-Synthes, Paoli, PA, USA) or 3.5-mm (Acumed, Hillsboro, OR, USA) LCP-based ulna osteotomy system, the dedicated surgical system for guided USOs. All osteotomies were performed obliquely or transversely using an orthopedic oscillating saw around the distal one third of the ulna, followed by plate fixation. For preventing heat injury to the osteotomized ulna during surgery, we stopped the sawing intermittently and cooled the osteotomy site by continuous cold saline irrigation in both groups [16]. We prioritized maintaining the inborn longitudinal and rotational alignment of the ulna over minimizing the osteotomy reduction gap during reduction osteotomy. Even in a non-paralleled osteotomy, we performed reduction by moving the distal segment proximally along the longitudinal axis to avoid angulated or rotated ulna after reduction osteotomy. Regardless of the two different osteotomy techniques, we performed arthroscopy for all wrists before embarking on USO. The arthroscopy aimed to confirm the UIS and to treat degeneration in the ulnocarpal joint. The procedure did not differ between the two groups. We made 3-4 portal to use as a viewing portal and 6R portal as a working portal. The degenerative tissues were debrided by an arthroscopic shaver and pinch [6, 17]. After surgery, both groups followed the same protocol for postoperative rehabilitation. Tolerable active wrist and forearm movements were allowed after placing the wrists in a short-arm splint for 4 weeks.

### Background data collection

**Fig. 3:**
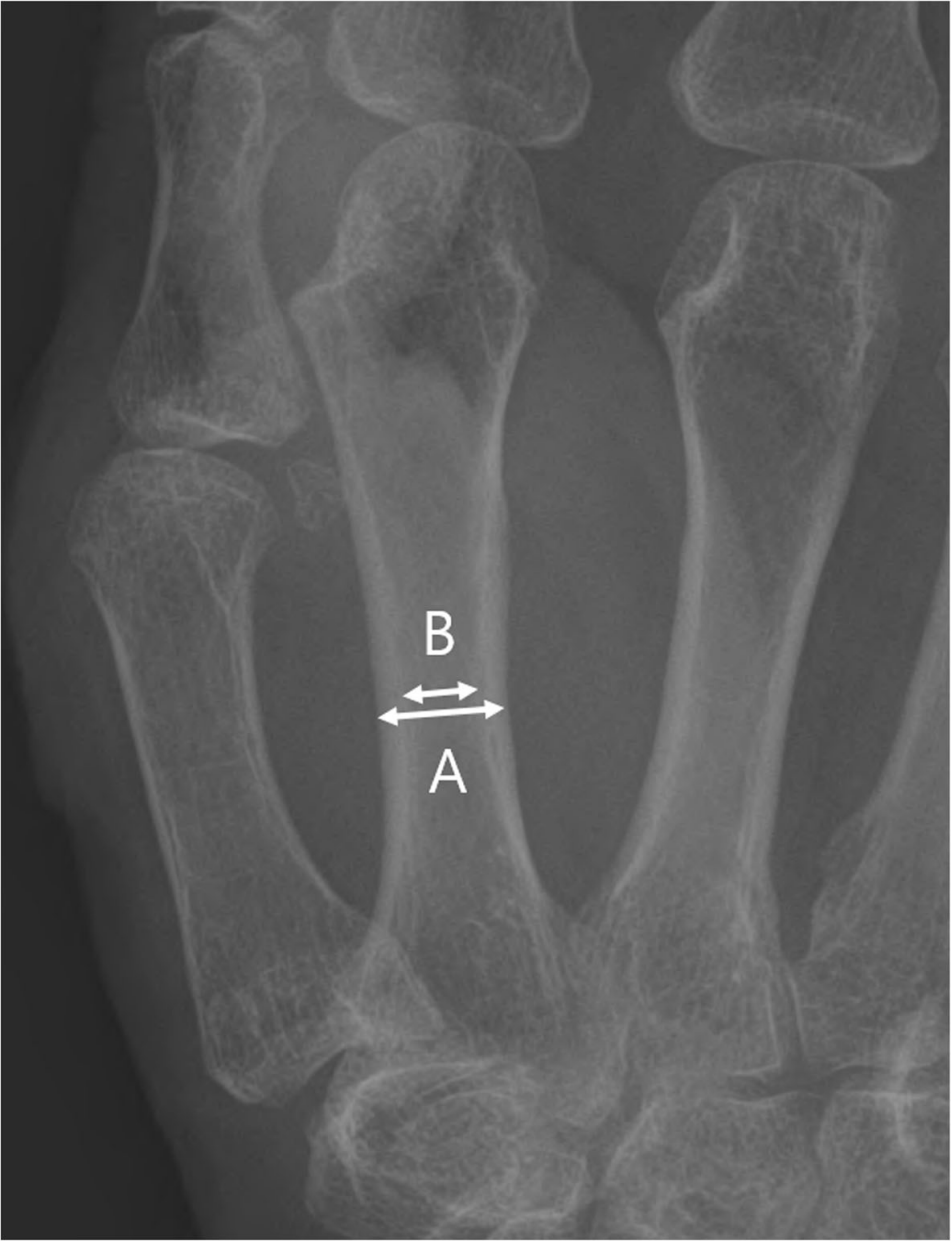
The second metacarpal cortical percentage (2MCP) for assessment of the bone quality. 2MCP is calculated by the formula [(**a**, **b**)/A] × 100 (**a** outer diameter of the second metacarpal isthmus, **b** inner diameter of the second metacarpal isthmus)

The patients” background data, including sex, age, affected side, body mass index (BMI), and bone quality (second metacarpal cortical percentage: 2MCP), were recorded [18]. BMI was calculated using the patients’ height and weight as BMI = weight [kg]/height [m]^2^ [19]. Patients with severe obesity (BMI ≥ 30 kg/m^2^) were excluded from this study [19, 20]. For bone quality assessments, we calculated the 2MCP from true posterior-anterior views of the preoperative hand or wrist radiographs [18]. All simple radiographs were taken on normal films (distance, 110 cm; power, 57 KV). The narrowest point (isthmus) of the second metacarpal shaft was focused and magnified to optimize visualization with the picture archiving and communication system (PiView STAR, Infinitt Healthcare Co. Ltd, Seoul, South Korea), a reliable method for orthopedic measurements [21, 22]. The transverse diameter of the isthmus of the second metacarpal bone was measured (A) [18]. Then, parallel measurements were made of the cancellous or intramedullary component at the same location (B). We used the formula [(A—B)/A] × 100 to calculate the 2MCP (Fig. 3) [18]. All variables were measured twice every 2 weeks by two independent orthopedists to avoid intra- or inter-observer errors, and the mean was calculated for each patient. Patients with 2 MCP < 50% were considered to have a higher likelihood of osteoporosis and were excluded from this study [18]. Complications associated with osteotomy healing, such as metal failure, screw loosening, delayed union, nonunion, and refracture after plate removal, were determined.

### Data collection on healing after osteotomy

We divided the healing process into osteotomy union and consolidation. Osteotomy union was defined by a callus formation bridging all cortices of the four planes observed on simple radiographs and no pain at the osteotomy site in the manual stress test. Osteotomy consolidation was defined by the disappearance of any trace at the osteotomy site in all four planes of simple radiographs [9, 15]. A union achieved after 6 months was considered a delayed union; when no union occured within 6 months and no radiographic improvements occurred during 3 consecutive months, it was defined as a nonunion [9]. Four plane simple radiographs (posteroanterior, lateral, internal, and external oblique views) were taken monthly until osteotomy union was achieved and every 3 months until osteotomy consolidation was completed to determine the length of the osteotomy reduction gap, the time to osteotomy union, and the time to complete consolidation for all the wrists. For protecting patients and radiographers from radiation, all simple radiographs were taken under radiation protection with a lead apron and thyroid protector, and no more radiographs were taken after achieving complete osteotomy consolidation. Two orthopedists blindly determined these values, twice every 2 weeks, and the average for each wrist was calculated.

### Data collection on surgical factors affecting the healing process

We recorded the direction of osteotomy, length of ulnar shortening, usage of a lag screw, and plate type used in each wrist to analyze the surgical factors affecting osteotomy healing. Based on the direction (an acute angle between the osteotomy plane and longitudinal axis of the ulna), the osteotomy was classified as transverse and oblique. The transverse osteotomy was defined as an angle of ≥ 60° between the osteotomy plane and longitudinal axis, while oblique osteotomy was defined by an angle < 60° (Fig. 2b). The ulnar variance was measured using the method of perpendicular in a true posterior-anterior simple radiograph of the wrist [23]. The length of the ulnar shortening was calculated as the difference between the preoperative and immediate postoperative ulnar variance. The lag screw usage was recorded as a “yes” or “no”, and the plate type used for osteotomy fixation was recorded as either “LC-DCP” or “LCP.” The osteotomy direction and length of ulnar shortening were also blindly measured twice every 2 weeks by two orthopedists and the average for each wrist was calculated.

### Sample size and statistical analysis

#### Power analysis

Using data from the study by Gaspar et al. [9], a prior power analysis was performed to determine the sample size needed to detect a difference in time to osteotomy union between freehand and guided osteotomies using the *t* test and Pearson’s chi-square test. Assuming a normal distribution and effect size of 1, we needed to enroll a minimum of 62 wrists in each group to detect a significant difference with 80% power (*α* = 0.05, *β* = 0.2). The sample size was calculated using the G*Power program (version 3.1.9).

### Statistical analysis

For confirming that there were no differences in patients’ background characteristics between the groups 1 and 2, a *t* test was performed for age and BMI, and Pearson’s chi-square test for sex, affected side, the direction of osteotomy, use of a lag screw, and type of plate used. The Mann-Whitney test was performed for the 2MCP and length of ulnar shortening. To compare the time to osteotomy union and complete consolidation, and the osteotomy reduction gap between the two groups, the Mann-Whitney test was also performed following Bonferroni correction (*α* = 0.05/4 = 0.0125).

Multiple regression tests were performed to identify the surgical factors affecting healing. The factors, as described in the “Study design” section, were considered as potential surgical factors associated with osteotomy healing. The cut-off length of the osteotomy reduction gap to obtain osteotomy union within 6 months and the maximal periods to achieve complete osteotomy consolidation were statistically calculated using receiver operating characteristic (ROC) curves. *P* < 0.05 was considered statistically significant in this regression test.

## Results

The patients were aged between 17 and 68 years (mean: 42). Of the patients, 78 were in men and 58 were in women. The patients were followed-up for an average of 73.5 (range: 29.7–160.7) months. Both groups matched well in terms of age, sex, affected side, BMI, and 2MCP (Table 1). There were 11 delayed unions in group 1 (11 of 74, 14.9%) and six in group 2 (6 of 62, 9.7%). Three of the 11 delayed unions in group 1 (freehand osteotomy) and one of six delayed unions (guided osteotomy) were accompanied by screw loosening. There were two refractures in group 1 and one in group 2 after plate removal. However, osteotomy unions were achieved in all wrists by the last follow-up visit, without revision surgery.

**Table 1:** Comparisons of background characteristics between groups

	Group 1 (*n* = 74)	Group 2 (*n* = 62)	*p* value
Age^a^ (years)	40.45 ± 9.18	40.29 ± 12.03	0.932
Sex (male to female)	42:32	36:26	0.878
Affected side (right to left)	38:36	30:32	0.731
BMI^a^ (kg/m^2^)	23.89 ± 2.61	23.91 ± 3.08	0.970
2MCP^a^ (%)	66.72 ± 4.62	65.58 ± 4.49	0.320

^a^Continuous variables expressed as mean ± Standard deviation. Statistical analyses were performed using the *t* test for age and BMI, Pearson’s chi-square test for categorized variables, and Mann-Whitney test for 2MCP

*BMI* body mass index, *2MCP* second metacarpal cortical percentage

The osteotomy reduction gap was longer in group 1 than in group 2 (group 1 = 0.60 ± 0.64 mm; group 2 = 0.28 ± 0.14 mm; *p* = 0.002). The time to osteotomy union was longer in group 1 than in group 2 (group 1 = 5.11 ± 2.67 months, group 2 = 3.61 ± 1.97 months, *p* < 0.001), and the time to complete osteotomy consolidation was also longer in group 1 than in group 2 (group 1 = 20.24 ± 9.46 months; group 2 = 15.81 ± 6.60 months, *p* = 0.002) (Table 2).

**Table 2:** Comparisons of surgical technique and osteotomy healing between groups

	Group 1 (*n* = 74)	Group 2 (*n* = 62)	*p* value
Osteotomy direction (transverse to oblique)	18:56	32:30	0.001^**†**^
Use of lag screw (yes to no)	27:47	25:37	0.647
Plate type (LC-DCP to LCP)	60:14	0:62	< 0.001^**†**^
Length of ulnar shortening^a^ (mm)	3.08 ± 1.05	2.86 ± 1.47	0.070
Osteotomy reduction gap^a^ (mm)	0.60 ± 0.64	0.28 ± 0.41	0.002^**†**^
Time to union^a^ (months)M	5.11 ± 2.67	3.61 ± 1.97	< 0.001^**†**^
Time to consolidation^a^ (months)	20.25 ± 9.46	15.81 ± 6.60	0.002^**†**^

^a^Continuous variables expressed as mean ± standard deviation. Statistical analyses were performed using Pearson’s chi-square test for categorized variables and the Mann-Whitney test for continuous variables

*LC-DCP* limited contact dynamic compression plate, *LCP* locking compression plate

^**†**^Statistical difference between groups

**Fig. 4:**
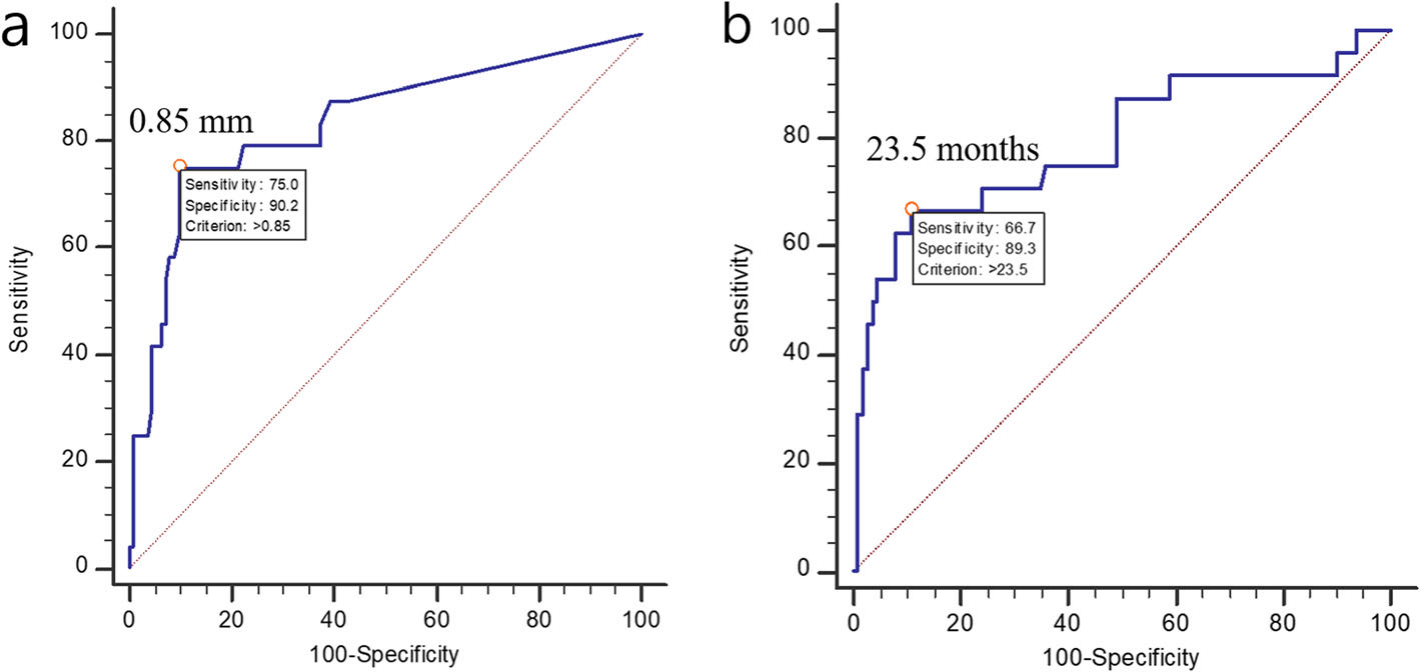
Receiver operating characteristic curves to predict the cut-off value for **a** the osteotomy reduction gap to obtain osteotomy union within 6 months (0.85 mm) and **b** the time to complete osteotomy consolidation to differentiate the failure of osteotomy consolidation (23.5 months)

The osteotomy reduction gap had a strong positive correlation with both time to osteotomy union (*p* < 0.001) and complete osteotomy consolidation (*p* < 0.001). The LC-DCP plate lengthened the time to consolidation (*p* < 0.001) (Tables 3 and 4). Statistically, the osteotomy reduction gap to predict timely osteotomy union (within 6 months) was 0.85 mm with 75% sensitivity and 90.2% specificity (area under the curve: 0.83; *p* < 0.001), and the time to complete consolidation of longer than 23.5 months had 66.7% sensitivity and 89.3% specificity for differentiating failure of osteotomy consolidation (area under the curve: 0.792; *p* < 0.001) (Fig. 4a). Therefore, surgeons should avoid osteotomy reduction gaps of > 0.85 mm for timely osteotomy union. Osteotomy that was not completely consolidated within 23.5 months was expected to have permanent consolidation failure (Fig. 4b).

**Table 3:** Univariate regression analysis of the surgical factors affecting osteotomy healing

Variable	Univariate regression analysis					
	Time to osteotomy union			Time to osteotomy consolidation		
	*R*	*t* value	*p* value	*R*	*t* value	*p* value
Osteotomy direction	0.002	0.500	0.618	0.00	0.171	0.864
Length of ulnar shortening	0.002	− 0.461	0.646	0.017	− 1.538	0.126
Use of lag screw	0.003	0.585	0.560	0.021	1.689	0.094
Plate type						
LC-DCP	0.047	1.033	0.303	0.124	2.910	0.004^**†**^
LCP	0.047	− 1.094	0.276	0.124	− 0.517	0.606
Osteotomy reduction gap	0.330	8.117	< 0.001^**†**^	0.175	5.324	< 0.001^†^

*R* coefficient of determination, *LC-DCP* limited contact dynamic compression plate, *LCP* locking compression plate

^**†**^Statistical difference between groups

**Table 4:** Multivariate regression analysis of the surgical factors affecting osteotomy healing

Variable	Multivariate regression analysis					
	Time to osteotomy union			Time to osteotomy consolidation		
	adjR	*t* value	*p* value	adjR	*t* value	*p* value
Length of ulnar shortening	–	–	–	0.280	-1.920	0.060
Use of lag screw	–	–	–	0.280	1.440	0.150
Plate type						
LC-DCP	–	–	–	0.280	3.270	< 0.001^**†**^
LCP	–	–	–	0.280	0.790	0.430
Osteotomy reduction gap	0.330	8.030	< 0.001^**†**^	0.280	4.180	< 0.001^**†**^

*adjR* adjusted coefficient of determination, *LC-DCP* limited contact dynamic compression plate, *LCP* locking compression plate

^**†**^Statistical difference between groups

## Discussion

The most popular osteotomy technique for USO was freehand transverse osteotomy [24]. However, the osteotomy union was slow and the incidence of nonunion was high using freehand transverse osteotomy since performing a precise and parallel osteotomy was challenging [25–27]. Rayhack et al. demonstrated a guided oblique osteotomy technique using an osteotomy jig for precise USO [26]. That resulted in a shorter union time than that with freehand transverse osteotomy [26]. They explained that the broader cross-section and lag screw usage in oblique osteotomy aided healing [26]. However, their explanations were theoretical and were not proven objectively. In addition, they did not consider the effects of patient-related factors on bone healing when comparing the two osteotomies [26].

This study excluded patients with patient-related factors to eliminate bias in analyzing the effects of osteotomy techniques on osteotomy healing. Similar to the previous studies, the osteotomy reduction gap, time to osteotomy union, and time to complete consolidation were shorter in guided than in freehand osteotomy [25–27]. That was because parallel surfaces for osteotomy could be obtained more easily with guided than with freehand osteotomy, and the parallel osteotomy surfaces enabled better osteotomy reduction and contact (minimal osteotomy reduction gap). Thirty-eight of 62 osteotomies (61.3%) in the group 2 had osteotomy reduction gaps of 0 mm, while only 28 of 74 osteotomies (37.8%) in the group 1 had osteotomy reduction gaps of 0 mm.

The effects of detailed surgical techniques on osteotomy healing have not been investigated yet since past studies only involved patients who underwent freehand transverse or guided oblique osteotomies with lag screws [27, 28]. In the present study, in contrast, the nine combinations of surgical techniques evaluated to statistically analyze the effects of surgical techniques on osteotomy healing were as follows: *(1) Manual transverse osteotomy + LC-DCP fixation: 16 wrists; (2) Manual transverse osteotomy + LCP fixation: 2 wrists; (3) Manual oblique osteotomy + LC-DCP fixation: 20 wrists*; *(4) Manual oblique osteotomy + LC-DCP fixation + Lag screw: 24 wrists; (5) Manual oblique osteotomy + LCP fixation: 9 wrists; (6) Manual oblique osteotomy + LCP fixation + Lag screw: 3 wrists; (7) Guided transverse osteotomy + LCP fixation: 32 wrists; (8) Guided oblique osteotomy + LCP fixation: 5 wrists;* and *(9) Guided oblique osteotomy + LCP fixation + Lag screw: 25 wrists.*

**Fig. 5:**
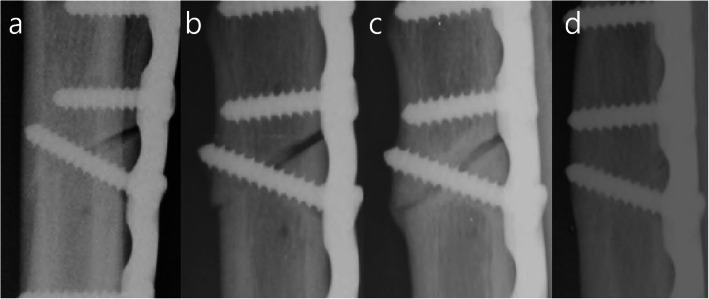
Serial radiographs of a freehand osteotomy with unparallel osteotomy surfaces and a long osteotomy reduction gap. **a** Immediate postoperative radiograph demonstrating that the lag screw could not compress a long osteotomy reduction gap. **b** After 2 months, bone defect still exists. **c** At 6 months, the osteotomy is united. **d** The complete osteotomy consolidation is achieved at 22 months

**Fig. 6:**
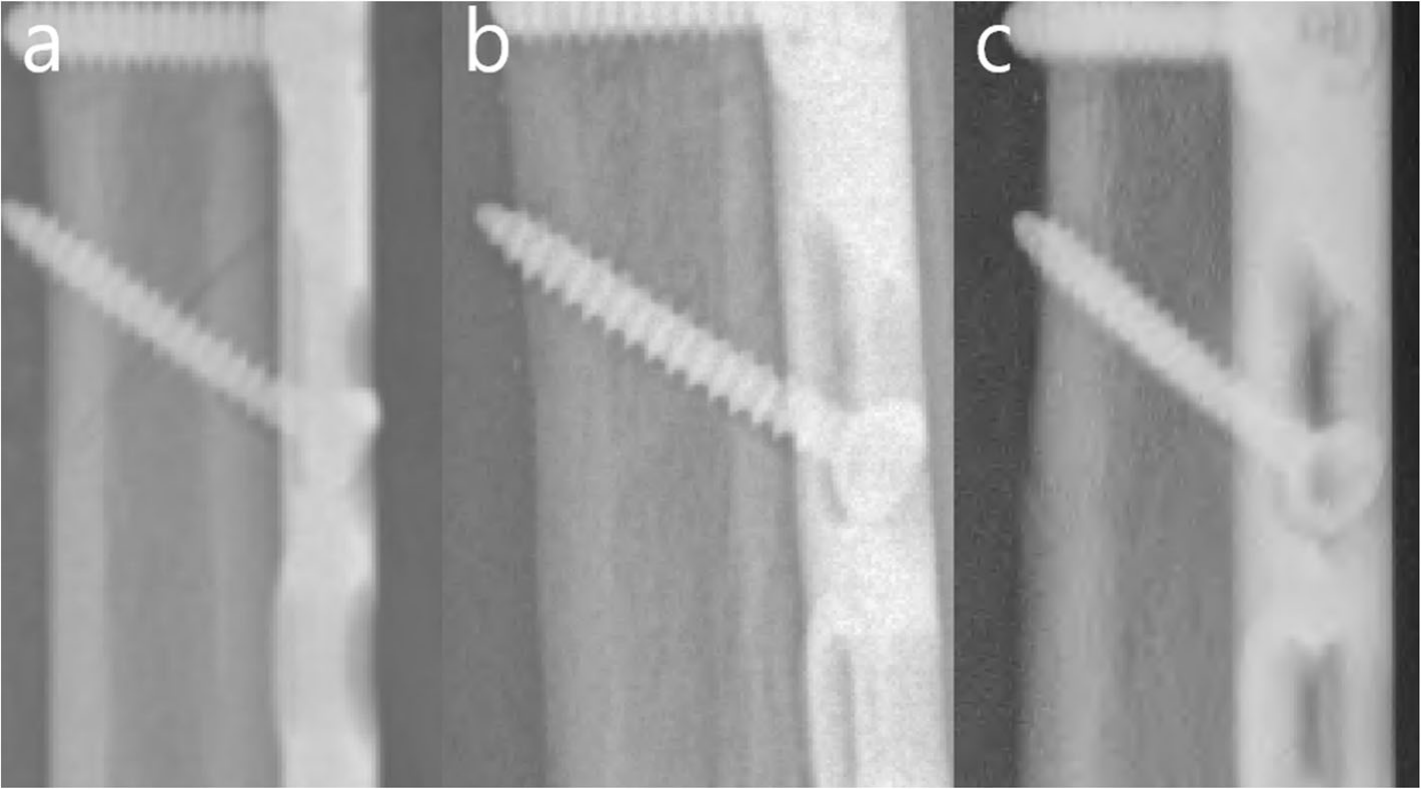
Serial radiographs of a guided osteotomy with paralleled osteotomy and minimal osteotomy reduction gap. **a** Immediate postoperative radiograph demonstrating that the lag screw could enhance the compression and stability of the osteotomy. **b** After 2 months, the osteotomy is united. **c** At 10 months, complete osteotomy consolidation is achieved.

Previous studies reported that osteotomy union was faster in oblique than in transverse osteotomy owing to the broad osteotomy surface and use of a lag screw in oblique osteotomy enhancing the compression, contact, and stability of the osteotomy [26–28]. However, our regression analysis revealed that only the osteotomy reduction gap affected osteotomy union and consolidation. The longer the osteotomy reduction gap, the longer the time to osteotomy healing regardless of other surgical factors (direction of osteotomy, usage of a lag screw, length of ulnar shortening, and type of plate used) in this study. The broad surface in oblique osteotomy was also not crucial for timely osteotomy healing since the time to osteotomy healing was similar to that in transverse osteotomy if the osteotomy reduction gap was minimal. In addition, the lag screw could not compress the osteotomy or increase the stability when the osteotomy reduction gap was long (Figs. 5 and 6). A long reduction gap can induce micromotion at the osteotomy site even after rigid fixation by LCP or LC-DCP, inhibiting the osteotomy healing [29–31].

The LC-DCP or LCP stabilized all osteotomies in this study. The plate type did not affect the time to union but affected the time to consolidation; the time to consolidation with LC-DCP > LCP. Though the LCP is more likely to maintain the osteotomy stability with a gap than the LC-DCP, both LC-DCP and LCP provide enough initial stability to withstand postoperative loads [32, 33], and the wrist immobilization and bone strength of healthy ulna assisted stability maintenance at the initial stage of osteotomy healing. Therefore, the plate type did not affect the time to osteotomy union. However, the LC-DCP delayed the osteotomy consolidation and the time to complete osteotomy consolidation with LC-DCP > LCP. The osteotomy reduction gap was longer in the LC-DCP than in the LCP since most of the freehand osteotomies were fixed by the LC-DCP. Moreover, The LC-DCP also has inferior long-term osteotomy stability with a longer gap [32, 34]. Therefore, the LC-DCP delayed the time to osteotomy consolidation. The length of the ulnar shortening did not affect osteotomy healing. This means that parallel osteotomy leads to better osteotomy reduction regardless of shortening length.

**Fig. 7:**
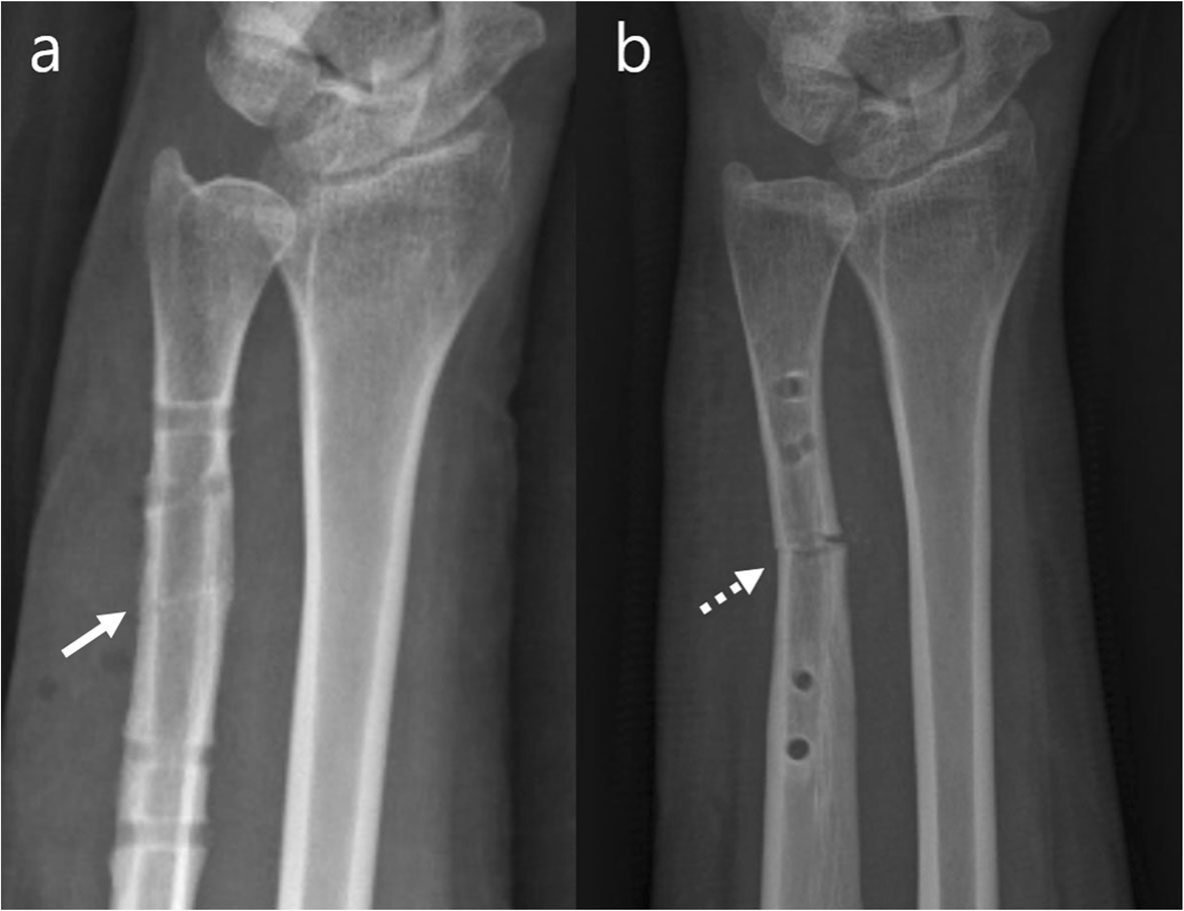
Radiograph of the case with **a** osteotomy trace (solid arrow) and **b** refracture after plate removal (dot arrow)

*Our statistical calculation with ROC curves revealed that a reduction gap of less than 0.85 mm is essential to obtain union within 6 months, and osteotomy traces that did not disappear within 23.5 months after osteotomy will persist permanently.* In contrast, most osteotomies on healthy individuals achieved complete consolidation within 23.5 months. For reduction gaps of longer than 0.85 mm, we recommend filling the gaps with the resected bones for better osteotomy healing. Otherwise, biological therapy using epithelial progenitor cell and mesenchymal stem-cell, physiologic therapy using intermittent pneumatic compression, low-intensity pulsed ultrasound, and shock wave, and pharmacological therapy using medication like teriparatide should be considered as an alternative treatment in that case [35–43]. In this study, all refractures occurred in the osteotomies with traces, while none occurred in the osteotomies without traces. The osteotomy trace represents incomplete consolidation. The reasonable time for plate removal is approximately 2 years after USO. If an osteotomy trace does not disappear even after 2 years, the plate should not be removed. Alternatively, for preventing refracture and diminishing the irritation, the thick plate should be replaced with a low-profile mini plate (Fig. 7).

There were 17 (12.5%) delayed unions in this study. All delayed unions caused pain during wrist motion, and the pain delayed a return to work. Four of them induced screw loosening. However, osteotomy unions were achieved in all delayed unions by the last follow-up visit, without revision surgery. The delayed union can cause symptoms and inhibit patients’ daily living. In a recent systematic review, the overall rate of nonunion among all osteotomy techniques was 4.0% [8]. Since the rate of delayed union is much higher than that of nonunion after USO, a delayed union can be a more frequent problem for patients who underwent USO. This study mainly aimed to overcome the delayed union of USO and provided the key point for that.

This study has some limitations. First, the effects of the saw blade on osteotomy healing were not estimated. A single blade saw of a 0.5-mm thickness was used during freehand and guided osteotomies using the 3.5 LCP osteotomy system. A double blade saw of 0.6 mm thickness was used in guided osteotomy with the 2.7 LCP osteotomy system. Heat injury can be severe in low-speed cutting with thicker blades and can cause irreversible bone damage [16]. However, it can be prevented by copious saline irrigation at the osteotomy site [44]. In this study, the authors stopped the sawing intermittently and cooled the osteotomy site by cold saline irrigation continuously to prevent heat injuries. In addition, the type of saw was a dependent surgical factor associated with the osteotomy technique and system. Therefore, it was inappropriate for our regression analysis. Second, we did not analyze the effects of the 2.7 and 3.5 LCPs on osteotomy healing separately since the number of 2.7 LCP was small and the present study aimed to compare the osteotomy healing between guided and freehand osteotomy. Finally, this study had a retrospective manner. We allocated the wrists to the two groups by the surgeon’s preference without randomization in this study. That can cause a selection bias. However, since both groups matched well in terms of the background data like age, sex, affected side, BMI, and 2MCP, our comparisons could be reliable.

## Conclusions

The minimal osteotomy reduction gap (< 0.85 mm) was crucial for timely osteotomy healing in the healthy ulna, and a guided osteotomy was beneficial for reducing the osteotomy reduction gap during USO. The trace of osteotomy indicates the failure of complete osteotomy consolidation. If there is any trace of osteotomy, the plate should not be removed to prevent refracture of the osteotomy.

## Data Availability

The datasets generated and/or analyzed during the current study are available in the Mendeley Data, [10.17632/vgj5x6nwvn.1].

## References

[1] Milch H (1941). Cuff resection of the ulna for malunited colles’ fracture. JBJS.

[2] Barbaric K, Rujevcan G, Labas M, Delimar D, Bicanic G (2015). Ulnar shortening osteotomy after distal radius fracture malunion: review of literature. Open Orthopaedics J..

[3] Tatebe M, Shinohara T, Okui N, Yamamoto M, Hirata H, Imaeda T (2012). Clinical, radiographic, and arthroscopic outcomes after ulnar shortening osteotomy: a long-term follow-up study. J Hand Surg.

[4] Clark SM, Geissler WB (2012). Results of ulnar shortening osteotomy with a new plate compression system. Hand..

[5] Sammer DM, Rizzo M (2010). Ulnar impaction. Hand Clin.

[6] Chun S, Palmer AK (1993). The ulnar impaction syndrome: follow-up of ulnar shortening osteotomy. J Hand Surg.

[7] Constantine KJ, Tomaino MM, Herndon JH, Sotereanos DG (2000). Comparison of ulnar shortening osteotomy and the wafer resection procedure as treatment for ulnar impaction syndrome. J Hand Surg.

[8] Owens J, Compton J, Day M, Glass N, Lawler E (2019). Nonunion rates among ulnar-shortening osteotomy for ulnar impaction syndrome: a systematic review. J Hand Surg Am.

[9] Gaspar MP, Kane PM, Zohn RC, Buckley T, Jacoby SM, Shin EK (2016). Variables prognostic for delayed union and nonunion following ulnar shortening fixed with a dedicated osteotomy plate. J Hand Surg.

[10] Brinker MR, O'Connor DP, Monla YT, Earthman TP. Metabolic and endocrine abnormalities in patients with nonunions. J Orthopaedic Trauma. 2007;21(8):557–70. 10.1097/BOT.0b013e31814d4dc6 17805023

[11] Giannoudis P, Tzioupis C, Almalki T, Buckley R (2007). Fracture healing in osteoporotic fractures: is it really different?: A basic science perspective. Injury..

[12] Hernandez RK, Do TP, Critchlow CW, Dent RE, Jick SS (2012). Patient-related risk factors for fracture-healing complications in the United Kingdom General Practice Research Database. Acta Orthopaedica..

[13] Patil M, Baseer H (2017). Obesity and fracture healing. Al Ameen J Med Sci..

[14] Richards CJ, Graf KW, Mashru RP (2017). The effect of opioids, alcohol, and nonsteroidal anti-inflammatory drugs on fracture union. Orthopedic Clin.

[15] Baek GH, Chung MS, Lee YH, Gong HS, Lee S, Kim HH (2005). Ulnar shortening osteotomy in idiopathic ulnar impaction syndrome. J Bone Joint Surg Am..

[16] Firoozbakhsh K, Moneim MS, Mikola E, Haltom S (2003). Heat generation during ulnar osteotomy with microsagittal saw blades. Lowa Orthopaedic J.

[17] Bickel KD (2008). Arthroscopic treatment of ulnar impaction syndrome. J Hand Surg..

[18] Schreiber JJ, Kamal RN, Yao J (2017). Simple assessment of global bone density and osteoporosis screening using standard radiographs of the hand. J Hand Surg..

[19] Region WWP, IASO I (2000). The Asia-Pacific perspective: redefining obesity and its treatment.

[20] Low S, Chin MC, Ma S, Heng D, Deurenberg-Yap M (2009). Rationale for redefining obesity in Asians. Ann Acad Med Singapore..

[21] Fowler JR, Ilyas AM (2011). The accuracy of digital radiography in orthopaedic applications. Clin Orthopaedics Related Res.

[22] Srinivasalu S, Modi HN, SMehta S, Suh S-W, Chen T, Murun T (2008). Cobb angle measurement of scoliosis using computer measurement of digitally acquired radiographs-intraobserver and interobserver variability. Asian Spine J..

[23] Bernstein DT, Linnell JD, Petersen NJ, Netscher DT (2018). Correlation of the lateral wrist radiograph to ulnar variance: a cadaveric study. J Hand Surg.

[24] Baek GH, Chung MS, Lee YH, Gong HS, Lee S, Kim HH (2006). Ulnar shortening osteotomy in idiopathic ulnar impaction syndrome: surgical technique. JBJS..

[25] Hulsizer D, Weiss A-PC, Akelman E (1997). Ulna-shortening osteotomy after failed arthroscopic debridement of the triangular fibrocartilage complex. J Hand Surg..

[26] Rayhack JM, Gasser SI, Latta LL, Ouellette EA, Milne EL (1993). Precision oblique osteotomy for shortening of the ulna. J Hand Surg..

[27] Sunil T, Wolff TW, Scheker LR, McCabe SJ, Gupta A (2006). A comparative study of ulnar-shortening osteotomy by the freehand technique versus the Rayhack technique. J Hand Surg..

[28] Köppel M, Hargreaves I, Herbert T (1997). Ulnar shortening osteotomy for ulnar carpal instability and ulnar carpal impaction. J Hand Surg..

[29] Perren SM (2002). Evolution of the internal fixation of long bone fractures: the scientific basis of biological internal fixation: choosing a new balance between stability and biology. The Journal of bone and joint surgery. Br Volume..

[30] Hente R, Lechner J, Fuechtmeier B, Schlegel U, Perren S (2001). Der Einfluss einer zeitlich limitierten kontrollierten Bewegung auf die Frakturheilung. Hefte Unfallchirurg..

[31] Perren S (2014). Fracture healing: fracture healing understood as the result of a fascinating cascade of physical and biological interactions. Part II.

[32] Florin M, Arzdorf M, Linke B, Auer JA (2005). Assessment of stiffness and strength of 4 different implants available for equine fracture treatment: a study on a 20° oblique long-bone fracture model using a bone substitute. Vet Surg.

[33] Uhl JM, Seguin B, Kapatkin AS, Schulz KS, Garcia TC, Stover SM (2008). Mechanical comparison of 3.5 mm broad dynamic compression plate, broad limited-contact dynamic compression plate, and narrow locking compression plate systems using interfragmentary gap models. Vet Surg..

[34] Alzahrani MM, Cota A, Alkhelaifi K, Harvey EJ. Mechanical evaluation of 2.7- versus 3.5-mm plating constructs for midshaft clavicle fractures. JAAOS. 2000;25(1):55–60. 10.5435/JAAOS-D-19-00495 32701682

[35] Andia I, Maffulli N (2017). Biological therapies in regenerative sports medicine. Sports Med..

[36] Coppola C, Del Buono A, Maffulli N (2015). Teriparatide in fracture non-unions. Transl Med UniSa..

[37] Oryan A, Alidadi S, Moshiri A, Maffulli N (2014). Bone regenerative medicine: classic options, novel strategies, and future directions. J Orthop Surg Res..

[38] Longo UG, Trovato U, Loppini M, Rizzello G, Khan WS, Maffulli N (2012). Tissue engineered strategies for pseudoarthrosis. Open Orthop J..

[39] Martinez de Albornoz P, Khanna A, Longo UG, Forriol F, Maffulli N (2011). The evidence of low-intensity pulsed ultrasound for in vitro, animal and human fracture healing. Br Med Bull.

[40] Keramaris NC, Kaptanis S, Moss HL, Loppini M, Pneumaticos S, Maffulli N (2012). Endothelial progenitor cells (EPCs) and mesenchymal stem cells (MSCs) in bone healing. Curr Stem Cell Res Ther..

[41] Furia JP, Rompe JD, Cacchio A, Maffulli N (2010). Shock wave therapy as a treatment of nonunions, avascular necrosis, and delayed healing of stress fractures. Foot Ankle Clin..

[42] Khanna A, Nelmes RT, Gougoulias N, Maffulli N, Gray J (2009). The effects of LIPUS on soft-tissue healing: a review of literature. Br Med Bull..

[43] Khanna A, Gougoulias N, Maffulli N (2008). Intermittent pneumatic compression in fracture and soft-tissue injuries healing. Br Med Bull..

[44] Rashad A, Sadr-Eshkevari P, Heiland M, Smeets R, Hanken H, Gröbe A (2015). Intraosseous heat generation during sonic, ultrasonic and conventional osteotomy. J Cranio-Maxillofacial Surg.

